# Isolation and functional characterization of primary human proximal tubular epithelial cells from brain‐dead organ donors

**DOI:** 10.14814/phy2.70963

**Published:** 2026-06-04

**Authors:** Daiki Aomura, Kevin G. Burfeind, Yoshio Funahashi, Tahnee Groat, Adam C. Munhall, Darren J. Malinoski, Michael P. Hutchens

**Affiliations:** ^1^ Department of Anesthesiology and Perioperative Medicine Oregon Health and Science University Portland Oregon USA; ^2^ Department of Emergency and Critical Care Nagoya University Hospital Nagoya Aichi Japan; ^3^ Department of Nephrology Nagoya University Hospital Nagoya Aichi Japan; ^4^ Department of Surgery Oregon Health and Science University Portland Oregon USA; ^5^ Division of Operative Care Portland VA Medical Center Portland Oregon USA; ^6^ Division of Nephrology and Hypertension Oregon Health and Science University Portland Oregon USA

**Keywords:** endocytosis, megalin, primary kidney culture, proximal tubule, vascular cell adhesion protein 1

## Abstract

Kidney proximal tubules (PT) utilize endocytosis to maintain body homeostasis. Effective PT repair mechanisms after acute kidney injury (AKI) are crucial to avoid the transition to chronic kidney disease (CKD). Cultured PT cells are often utilized to study these processes, but studies demonstrate loss of functional transporter expression in nearly all available cell lines. Here, we describe acquisition and culture of human PT cells isolated from the kidneys of brain‐dead donors (bddPTC) not suitable for transplantation and designated to research. The kidney cortex was digested, and the cells filtered through strainers were cultured. After passage 1, the cells were labeled for CD10 and CD13, and bddPTC were isolated via fluorescence‐activated sorting. bddPTC strongly expressed the brush border transport receptor megalin and aquaporin‐1 in bddPTC. Functionally, uptake of the megalin ligand myoglobin was greater in bddPTC than HK2, a human PT cell line. Pretreatment with the megalin inhibitor cilastatin, megalin‐targeted small interfering RNA, and endocytosis inhibitors reduced myoglobin uptake in bddPTC. Vascular cell adhesion protein 1, a marker for failed repair of PT after injury, was highly expressed in bddPTC. We provide reproducible means by which bddPTC may be used to study functional assessment and perhaps failed repair of PT.

## Graphical abstract

Protocol for isolation and culture of primary proximal tubular epithelial cells from kidneys retrieved from brain‐dead organ donors and designated for research after rejection for transplantation. These cells, bbd‐PTEC, express canonical proximal tubule epithelial markers and demonstrate functional transport. Interestingly, they express VCAM‐1, a marker for failed or maladaptive repair.

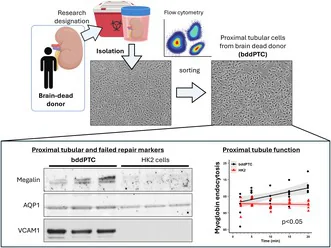

## 1 INTRODUCTION

The proximal tubule (PT) constitutes over 50% of the normal kidney volume, reabsorbing small proteins filtered through the glomeruli, including albumin and myoglobin (Chevalier, 2016; Edwards et al., 2022). Megalin, a receptor expressed in the PT brush border, binds to a variety of proteins, triggering cellular uptake through endocytosis (Eshbach & Weisz, 2017; Goto et al., 2023). Reabsorption of small proteins through megalin‐mediated endocytosis is a critical homeostatic function of the PT (Carone et al., 1979; Hoppensack et al., 2013). PT cells also play a significant role in kidney pathology, as they are the most susceptible part of the kidney to ischemia but also are capable of rapid functional regeneration (Chevalier, 2016). However, injury may be so severe that aberrant repair (termed “failed”, or “maladaptive” repair) occurs, which is thought to contribute to kidney fibrosis and transition to chronic kidney disease (CKD) (Chevalier, 2016; Wang & Zhang, 2022). Proximal tubule physiologic and pathophysiologic functions, including endocytosis and repair, are complex (Vesey et al., 2009). To study molecular mechanisms of PT function, a controlled environment is required, leading to a high need for physiologically functional cultured human PT cells (Vesey et al., 2009).

Although immortalized human PT cell lines are commercially available and widely used (Idrisova et al., 2022; Kaur & Dufour, 2012; Ryan et al., 1994), recent studies demonstrate that such cell lines lose critical biochemical and transport properties (Orosz et al., 2004; Prozialeck et al., 2006; Vesey et al., 2009). Ideally, a highly‐controlled in vitro system which recapitulates human in vivo function could be utilized. Despite the promising development of organoids and PT organs‐on‐a‐chip, no in vitro system currently maintains in vivo PT function. For example, organoids are cultured fully ex vivo and sourced from induced pluripotent stem cells with variable success in terminal differentiation (Montserrat et al., 2012; Vanslambrouck et al., 2023). Terminally differentiated human primary cells thus offer advantages for culture systems. However, human‐sourced PT cells have significant limitations. Human kidney cells can be isolated from living patients using the grossly normal‐appearing part of surgically resected malignant kidneys or from kidney biopsy specimens (Aceves et al., 2022; Petreski et al., 2023; Presnell et al., 2011; Vesey et al., 2009). However, human PT cells isolated from living patients with cancer are potentially contaminated with malignant cells and biopsy samples are limited in yield (An et al., 2023; Yaigoub et al., 2022). Moreover, this requires scheduling and cross‐organizational coordination, and studies performed on tissue from living donors are subject to additional ethical and legal compliance restrictions (Danačíková et al., 2024).

Here, we introduce a straightforward and reproducible protocol for isolation of PT cells from the kidneys recovered from brain‐dead donors (bddPTC) judged unsuitable for transplantation and subsequently designated for research. Our protocol does not involve living patients and does not include donor protected health information, and therefore received non‐human subjects research determination from our institutional review board. We provide methodologic detail, and we characterize bddPTC, demonstrating that they retain important in vivo PT functional characteristics while demonstrating a potentially important phenotypic difference.

## 2 MATERIALS AND METHODS

### 2.1 Isolation and culture of bddPTC

#### 2.1.1 Acquisition of human donor kidneys

Kidneys of brain‐dead donors were obtained from a local organ procurement organization if they were deemed unsuitable for transplantation, and the donor family provided authorization for research. Exclusion criteria included: age under 18 years old, lack of research authorization, positivity for antibody against hepatitis C or SARS‐CoV‐2 antigen. Organs were provided from the organ procurement organization to the receiving laboratory without any identifying information on the laboratory side. Under National Institutes of Health guidelines (https://grants.nih.gov/policy‐and‐compliance/policy‐topics/human‐subjects/research), research performed using such tissue is not human subjects research. The Oregon Health and Science University Institutional Review Board confirmed that research conducted using this tissue represents non‐human subjects research. Kidneys were processed in our laboratory within 24 h of organ recovery. Kidneys were flushed and stored in University of Wisconsin solution (Servator B, Global Transplant Solutions, Spartanburg SC, USA) immediately after recovery from the donor and were subsequently processed in our laboratory within 24 h of organ recovery. Kidneys from a total of 4 donors were analyzed in this study.

#### 2.1.2 Preparation of reagents and instruments for kidney dissection

1. Prepare the instruments and reagents listed in Table S1. Dulbecco's Modified Eagle Medium/Nutrient Mixture F‐12 (Cat. No. D8437, Sigma, St Louis, Mo) supplemented with Penicillin–Streptomycin 10,000 U/mL (Cat. No. 15140‐122, Thermo Fisher Scientific, Waltham, MA) (DMEM/F12‐PS) is the basic medium for the experiments in this study. Liberase™ (Cat. No. 5401119001, Sigma) dissolved in DMEM/F12‐PS at 0.26 U/mL is prepared as “digestion buffer” for the kidney tissue. DMEM/F12‐PS with 10% fetal bovine serum (FBS) (Cat. No. F‐0500‐A, Atlas Biologicals, Fort Collins, CO) or 1% FBS and 0.1% ITS Premix Universal Culture Supplement (Cat. No. 354351, Corning) are prepared as “culture medium‐10%FBS” and “culture medium‐1%FBS”, respectively.
2. Rat Collagen Type 1 (Cat. No. C3867, Sigma) is dissolved in 50 mL of ddH_2_O. Pipette the solution into ten T‐75 flasks (5 mL/flask). Gently shake the flasks to make the solution cover the bottom surface of the flask.
3. Incubate the flasks for 1 h at room temperature.
4. Remove the collagen solution. Open the lid and leave the flask in the biosafety hood for at least 1 h for drying.
5. Close the lid and keep the flasks in the incubator at 37°C.
6. Warm digestion medium, culture medium‐10%FBS, and culture medium‐1%FBS at 37°C in water bath.
7. Prepare the instruments, including a sterilized tray, beaker, blade, and forceps. Place the tray on ice, and place the other instruments in the tray for cooling.

#### 2.1.3 Dissection/cell suspension

Perform all kidney dissection and cell isolation procedures in a biosafety hood. Operators must wear personal protective equipment and use double gloves.

1. Place the donor kidney on the tray on ice.
2. Spray the outside of the kidney with 75% ethanol solution to reduce bacterial contamination.
3. Remove the perirenal fat and renal capsule using a blade and forceps
4. Divide the kidneys coronally in half and expose the renal papilla.
5. Dissect the cortex, about 10 mm from the surface, and cut into 1.5 cm pieces.
6. Put the cut kidney pieces in the beaker cooled on the tray, rinse with 20 mL of cold phosphate buffered saline (PBS) at pH 7.2.
7. If desired, after rinsing, place several pieces in 4% paraformaldehyde (PFA) for fixation and histological assessment.
8. Mince remaining pieces with forceps until homozygous.
9. Transfer the minced/homozygous tissue to ten 50 mL falcon tubes and fill each tube to 30 mL with cold PBS.
10. Wait for the tissue to naturally settle to the bottom of the tube (for 1 or 2 min without centrifugation) and then remove the supernatant.
11. Repeat the step (rinsing with cold PBS and removing supernatant) 1–3 times until the supernatant becomes transparent.
12. Add 20 mL digestion medium to each tube and incubate at 37°C for 2 h while gently shaking.
13. Add 20 mL of culture medium‐10%FBS in each tube to stop tissue digestion
14. Set a 27 cm diameter, 250 μm cell strainer (SKU. sz0000169, QuaFooLAB purchased through Amazon.com, Seattle, WA) on a new sterilized dish. Transfer the tissue in all tubes onto the strainer and push the tissue using the syringe plunger while adding 50 mL of cold PBS.
15. Set 100 μm cell strainers (Cat. No. 352360, Corning, New York, NY) on five new 50 mL falcon tubes. Put a 250 μm filtered‐tissue solution on the strainer while adding 20 mL of cold PBS to each tube. Grind the tissue on the strainer using a syringe plunger if the strainer gets clotted.
16. Centrifuge the filtered solution at 500 G for 5 min.
17. Remove the supernatant and resuspend it in 20 mL of cold PBS to each tube.
18. Set 70 μm cell strainers (Cat. No. 352350, Corning) on five new 50 mL falcon tubes and pipette tissue suspension through strainer while adding 20 mL of cold PBS to each tube. Do not grind the tissue on 70 μm strainer.
19. Centrifuge the filtered solution at 500 G for 5 min.
20. Remove the supernatant and resuspend the pellet in each tube with 15 mL of culture medium‐10%FBS.
21. Put the resulting kidney cell (bddKC) single cell suspension in each tube to a collagen coated flask. Incubate overnight.

#### 2.1.4 Primary culture and cell sorting of bddKC

1. Replace the medium with culture medium‐1%FBS on the first full day of culture.
2. Change the medium every 2 or 3 days.
3. Cells should become confluent on day 5 or 6 of culture. When confluent, passage the cells using Trypsin–EDTA and continue culturing.
4. When bddKC become confluent at passage 1, trypsinize cells and transfer to round bottom‐96 well cell culture plate.
5. Centrifuge 5 min at 500 G at 4°C. Remove supernatant and reconstitute cell pellet with phycoerythrin‐conjugated anti‐CD10 antibody (1:100 dilution, Cat. No. 50‐166‐673, Thermo Fisher Scientific) and allophycocyanin‐conjugated anti‐CD13 antibody (1:200 dilution, Cat. No. 50‐165‐739, Thermo Fisher Scientific) in PBS.
6. Incubate at 4°C for 30 min. Avoid light from this point onward.
7. Centrifuge at 500 G at 4°C for 5 min. Remove supernatant.
8. Reconstitute the pellet with 150 μL of cold culture medium‐1%FBS.
9. Centrifuge at 500 G at 4°C for 5 min. Remove supernatant.
10. Reconstitute the pellet with 150 μL of cold Culture media‐1%FBS.
11. Add 2 μL of 4′,6‐diamidino‐2‐phenylindole (DAPI) solution (1:75 dilution, Cat. No. BDB564907, Thermo Fisher Scientific).
12. Sort CD10/CD13 double positive bddKC cells (van der Hauwaert et al., 2013).
13. Seed sorted cells (now “bddPTC” cells) on new T‐75 flask at 10^7^cells/ flask with culture medium‐1%FBS.
14. Culture bddPTC and use for experiments at passage 4 or less.

### 2.2 Assessment of cellular uptake of myoglobin

To determine the functional characteristics of bddPTC, myoglobin uptake was assessed and compared to that of HK2 cells, an established human PT cell line (Ryan et al., 1994). HK2 cells were purchased from the American Type Culture Collection (Manassas, VA). bddPTC and HK2 cells were cultured with culture medium‐1%FBS and insulin‐transferrin‐selenium mixture (ITS) (Cat. No. 354351, Corning, Corning, NY) (hereafter referred to as “culture medium”) for 24 h, followed by incubation with DMEM/F12 without FBS and ITS (“serum free medium”) for 24 h. Cells (*n* = 6 wells for each cell type) were incubated with serum free medium containing 5 μg/mL (0.3 μM) of fluorescein isothiocyanate‐conjugated myoglobin (FITC‐Mb) for 2, 5, 10, 15, or 20 min. 5 mg/mL. Myoglobin (Cat. No. M0630, Sigma) was conjugated to FITC (Cat. No. 12352200, Sigma) according to the manufacturer's instructions. After incubation with Hoechst 33342 (1:2000 dilution, Cat. No. 62249, Thermo Fisher Scientific) for 10 min, the fluorescence of each well for FITC (480–530 nm) and Hoechst (405–460 nm) was measured using a fluorescence microplate reader (VICTOR NIVO, Perkin Elmer, Waltham, MA), and the ratio of FITC and Hoechst signal was calculated as fluorescence of the cells. Representative fluorescence images were taken using an epifluorescence microscope (BZ‐X810, Keyence corporation, Osaka, Japan) and BZ‐X800 viewer with OP‐87767 filter cubes for Hoechst and FITC.

### 2.3 Knockdown of *LRP2* by small interfering RNA

To further assess endocytic function in bbdPTC, we used small interfering RNA (siRNA) directed at *LRP2*, which codes for megalin, a multi‐ligand endocytic receptor expressed by PT that plays a pivotal role in recovery of filtered proteins (Eshbach & Weisz, 2017; Goto et al., 2023). To induce megalin knockdown, bddPTC were incubated with reduced serum medium (OPTI‐MEM, Cat. No. 31985062, Thermo Fisher Scientific) containing Lipofectamine 3000, a lipofection reagent (1:20 dilution, Cat. No. 3000015, Thermo Fisher Scientific), and 60 pg/mL siRNA targeting *LRP2* (Cat. No. 4392420, Thermo Fisher Scientific) or scramble RNA (Cat. No. 4390844, Thermo Fisher Scientific) for 24 h. HK2 cells were treated with scramble RNA. To verify knockdown, quantitative real‐time polymerase chain reaction (qRT‐PCR) was utilized. RNA was extracted (Cat. Nos. 79656 and 74106, QIAGEN, Hilden, Germany) according to the manufacturer's instructions. 150 ng of RNA were reverse‐transcribed using Maxima First Strand cDNA Synthesis Kit (Cat. No. K1642, Thermo Fisher Scientific). Relative mRNA amounts were quantified using Luna® Universal qPCR Master Mix (Cat. No. N3003, New England Biolabs, Ipswich, MA) and a Step One Plus Real‐Time PCR System (Applied Biosystems, Carlsbad, CA). *LRP2* expression was quantified using the ΔΔCT method and normalized to *GAPDH* expression. Probes were purchased from Thermo Fisher Scientific (*LRP2*: Hs00189742_m1, *GAPDH*: Hs04420697_g1). *LRP2* mRNA expression in bddPTC treated with *LRP2* siRNA and HK2 cells treated with scramble RNA were normalized to *LRP2* expression in scramble RNA‐treated bddPTC.

### 2.4 Treatment of bddPTC with cilastatin, siRNA targeting *LRP2* , and endocytosis inhibitors

bddPTC were incubated for 24 h with culture medium containing 2, 20, or 200 mg/mL cilastatin (Cat. No. SML1283, Sigma, a megalin inhibitor (Hori et al., 2017)), 60 pg/mL siRNA targeting *LRP2*, 10 μM hydroxydynasore (Cat. No. 1256493‐34‐1, Sigma, an endocytosis inhibitor (Kirchhausen et al., 2008)), or 100 μM prochlorperazine (Cat. No. 5374‐32‐3, Sigma, an endocytosis inhibitor (Chew et al., 2020; Daniel et al., 2015)). Cells incubated with only culture medium were considered the vehicle group. After washing with PBS, cells were incubated with FITC‐Mb or vehicle and myoglobin uptake was assessed as described above. For the experiments with siRNA targeting *LRP2*, dynasore, and prochlorperazine, cells were incubated with FITC‐Mb or vehicle for 180 min.

### 2.5 Immunoblot

bddKC, bddPTC, and HK2 cells were incubated and lysed with radio‐immunoprecipitation assay lysis buffer at pH 7.5 supplemented with cOmplete Mini EDTA‐free protease inhibitors (Cat. No. 11836170001, Roche Applied Science, Mannheim, Germany). Concentrations of extracted protein were determined using a bicinchoninic acid protein assay kit (Cat. No. 23225, Thermo Fisher Scientific). Protein was denatured with 5 mM of dithiothreitol and alkylated with 12.5 mM iodoacetamide. Proteins were loaded onto gradient precast gels (Cat. No. NP0321, Thermo Fisher Scientific), electrophoresed, and transferred to polyvinyl difluoride membranes (Cat. No. LC2005, Thermo Fisher Scientific) using a mini–Gel Tank (Cat. No. A25977, Sigma). After blocking with 5% bovine serum albumin (Cat. No. 9048‐46‐8, Sigma), membranes were incubated overnight with primary antibodies against LRP2 (1:1000 dilution, Cat. No. PA5‐67900, Thermo Fisher Scientific), aquaporin‐1 (AQP1) (1:2000 dilution, Cat. No. PA5‐77842, Thermo Fisher Scientific), Zonula Occludens‐1 (ZO‐1) (1:1000 dilution, Cat. No. Z0750, USBiological, Swampscott, MA), or VCAM1 (1:1000 dilution, Cat. No. 134047, Abcam, Cambridge, UK) at 4°C, followed by incubation with horseradish peroxidase‐conjugated anti‐rabbit IgG (Cat. No. 31462, Thermo Fisher Scientific). Membranes were incubated with SuperSignal West Pico PLUS (Cat. No. 34580, Thermo Fisher Scientific) and protein signal detection was performed with ChemiDoc MP Bioimaging System (Bio‐Rad, Hercules, CA). The positions of the protein bands were determined by PageRuler™ Plus Pre‐stained Protein Ladder (Cat. No. 26619, Thermo Fisher Scientific) or HiMark™ Pre‐stained Protein Standards (Cat. No. LC5699, Thermo Fisher Scientific). For quantification, total protein was semi‐quantified using the Coomassie stain image of the same membrane, and the specific protein quantity was assessed as the ratio of the specific band density to total Coomassie density (Zhai et al., 2022; Li & Shen, 2013). The protein abundance of each group was described as relative change to that of bddPTC. Band intensities were measured densitometrically using NIH ImageJ software (National Institutes of Health, Bethesda, MD, USA).

### 2.6 Immunocytochemistry

bddPTC at passage 2 and 3 were cultured on 96‐well plates and fixed with 4%PFA for 10 min. Cells were blocked with 10% normal donkey serum (Cat. No. S30‐M, Sigma) for 30 min, followed by incubation in primary antibody against LRP2 (1:200 dilution, Cat. No. 76969, Abcam) or AQP1 (1:100 dilution, Cat. No. PA5‐77842, Thermo Fisher Scientific) and secondary antibodies (Cat. No. A‐32790 and A‐31572, Thermo Fisher Scientific) for 1 h, respectively. Cellular nuclei were stained with Hoechst 33342 (Cat. No. H‐3570, Thermo Fisher Scientific). Fluorescence images were taken using a Keyence BZ‐X810 microscope and BZ‐X800 viewer with OP‐87767 filter cubes for Hoechst, Alexa Fluor™ 488, and Alexa Fluor™ 555.

### 2.7 Periodic acid‐Schiff staining and immunohistochemistry

Human donor kidney sections were dissected, then fixed in 4%PFA, paraffin embedded, and sectioned into 6 μm slices with a microtome. Deparaffinized sections were stained with periodic acid‐Schiff (PAS). Heat induced‐epitope retrieval reaction was performed using Citrate‐Based Antigen Unmasking Solution (Cat. No. H‐3300, Vector laboratory, Newark, CA) and microwaves for immunohistochemistry. After blocking with 10% normal donkey serum, sections were incubated with primary antibody for LRP2 (1:2000 dilution, Cat. No. 76969, Abcam) or VCAM1 (1:800 dilution, Cat. No. 134047, Abcam) overnight at 4°C. Horseradish peroxidase‐conjugated goat anti‐rabbit IgG (Cat. No. 31462, Thermo Fisher Scientific) was used as secondary antibody and ImmPACT DAB Peroxidase Substrate Kit (Cat. No. SK‐4105, Vector laboratory) was used for visualization. The sections were counterstained with hematoxylin (Cat. No. 220365, Abcam). Slides were imaged at low and high power with a Keyence BZ‐X810 fluorescence microscope.

### 2.8 Flow cytometry analysis

bbdKC were thawed in DMEM medium, washed once in DMEM medium, then incubated for 30 min at 4°C with the following antibody mixture: allophycocyanin (APC) anti‐human VCAM1 (1:100 dilution, Cat. No. 305810, clone STA, BioLegend, San Diego, CA), phycoerythrin (PE) anti‐human CD10 (1:100 dilution, Cat. No. 312204, clone HI10a, BioLegend), live/dead fixable aqua (1:200 dilution, Cat. No. L34966, Thermo Fisher). Cells were then washed twice in DMEM medium and taken to the OHSU Flow Cytometry Core for analysis. Flow cytometry was performed using a BD FACSymphony flow cytometer. Data were analyzed with FlowJo v10 software (FlowJo LLC, Ashland, OR). Fluorescence minus one (CD10 and live/dead antibody mixture without VCAM1 antibody) was used for negative control to identify VCAM1 positive cells.

### 2.9 Reanalysis of mRNA expression in human cell lines

We reanalyzed publicly‐available data provided by the National Institutes of Health's Epithelial Systems Biology Laboratory (ESBL) (https://esbl.nhlbi.nih.gov/JBrowse/KCT/) (Khundmiri et al., 2021). *LRP2* and *VCAM1* mRNA quantifications in human PT cell lines including HK2, HKC11, HKC8, HPTC‐05‐CLA, and HPTC‐05‐LTR cells were retrieved and expression levels were standardized to the arithmetic mean of all 5 human PT cell lines. *LRP2* and *VCAM1* mRNA expressions were compared to expression in HK2 cells.

### 2.10 Statistical analysis

The ratio of LRP2‐positive and AQP1‐positive bddPTC at passage 2 and 3 was quantified using immunocytochemistry images. Two 10x images were assessed per replicate. Positive cells for LRP2, AQP1, and Hoechst (used to determine the total cell counts) were automatically identified using Cell Profiler software (Stirling et al., 2021). To assess FITC‐Mb uptake, fluorescent value at each time point was standardized to base fluorescence (fluorescence at 2 min after incubation) for each well. The difference in fluorescence slope between bddPTC and HK2 cells at different incubation time points was assessed as group interaction using a linear regression model. In experiments with cilastatin, fluorescent values at 15 min after incubation were compared using one‐way analysis of variance (ANOVA) followed by Dunnet's post‐hoc test. In *LRP2* siRNA and drug‐inhibition experiments, fluorescence value was standardized to the averaged fluorescence value of vehicle‐treated cells at the same time point. The difference in fluorescent change between the groups was assessed as the interaction of the groups with incubation time using repeated measurement (RM)‐ANOVA. For mRNA and protein expression experiments, differences between groups were assessed using one‐way ANOVA followed by Tukey's post‐hoc test. *LRP2* and *VCAM1* mRNA levels in human PT cell lines from the ESBL dataset were compared using one‐way ANOVA followed by Dunnet's post‐hoc test. Statistical analyses and graph creation were performed using the R software (version 4.5.1, R Foundation for Statistical Computing, Vienna, Austria).

## 3 RESULTS

### 3.1 Isolation and culture of bddPTC

**FIGURE 1:**
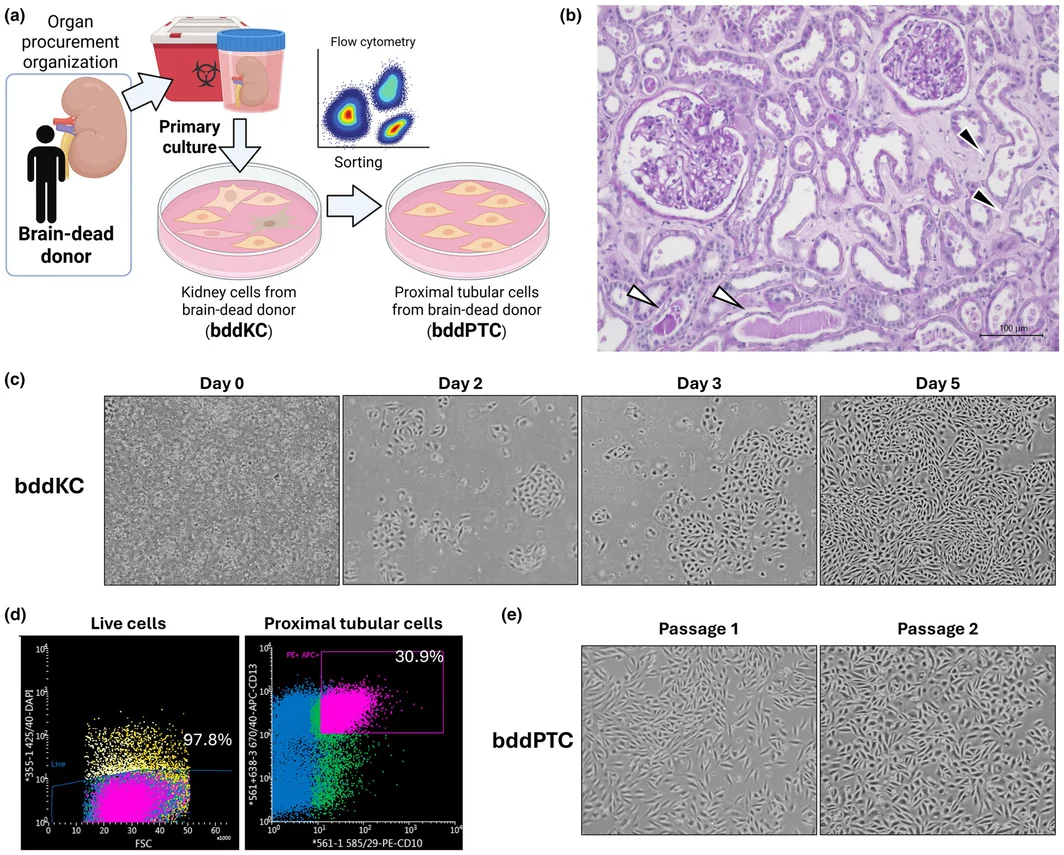
Isolation and culture of human proximal tubular epithelial cells. (a) Schematic of isolation of proximal tubular cells from brain‐dead donor (bddPTC). Kidney cells are primary cultured from the kidney of brain‐dead donors (bddKC). bddPTC are isolated from bddKC at passage 1 or 2 using a flow cytometer. (b) Representative Periodic acid‐Schiff staining of donor kidney. Acute tubular necrosis is evident, as well as tubular cell lining disruption (black arrow), brush border loss, and intratubular (white arrow). Magnification: × 20. Scale bar: 100 μm. (c) Phase‐contrast microscopic appearance of primary cultured bddKC. Magnification: × 4. Scale bar: 300 μm. (d) Representative bddKC flow cytometry cell. Approximately 95% of cells were live (4′,6‐diamidino‐2‐phenylindole (DAPI) negative) (left panel). bddPTC, identified as live, CD10 + CD13+ cells, were approximately 30% of all cells (right panel). (e) Phase‐contrast microscopic appearance of bddPTC at passage 1 and 2. Magnification: × 4. Scale bar: 300 μm.

A schematic of acquisition, isolation and culture of bddPTC from donor kidney is shown in Figure 1a. On gross examination, solitary renal cysts were the only frequent anatomic findings in donor kidneys. PAS‐stained histologic sections demonstrated frequent dilated/effaced proximal tubules and debris in the tubular lumen, indicative of moderate acute tubular necrosis. Tissue architecture otherwise was intact (representative image, Figure 1b). Immunohistochemistry indicated sustained expression of LRP2 in the PT brush border of the donor kidney (Figure S1). Following kidney dissociation and cell suspension, examination of cultured cells (bddKC) demonstrated non‐adherent cells and debris on the day of isolation (Figure 1c). However, bddKC were adherent in clusters by day 2 and became 100% confluent by day 5 of culture, demonstrating epithelioid shape. At the time of sorting, 95% of bddKC were live, and ~30% were CD10^+^/CD13^+^, which will be referred to as bddPTC hereafter (Figure 1d). At passages 1 and 2, sorted bddPTC demonstrated fusiform shape and rapid growth to confluence (Figure 1e).

### 3.2 bddPTC express megalin, AQP1, and ZO‐1 and take up myoglobin

**FIGURE 2:**
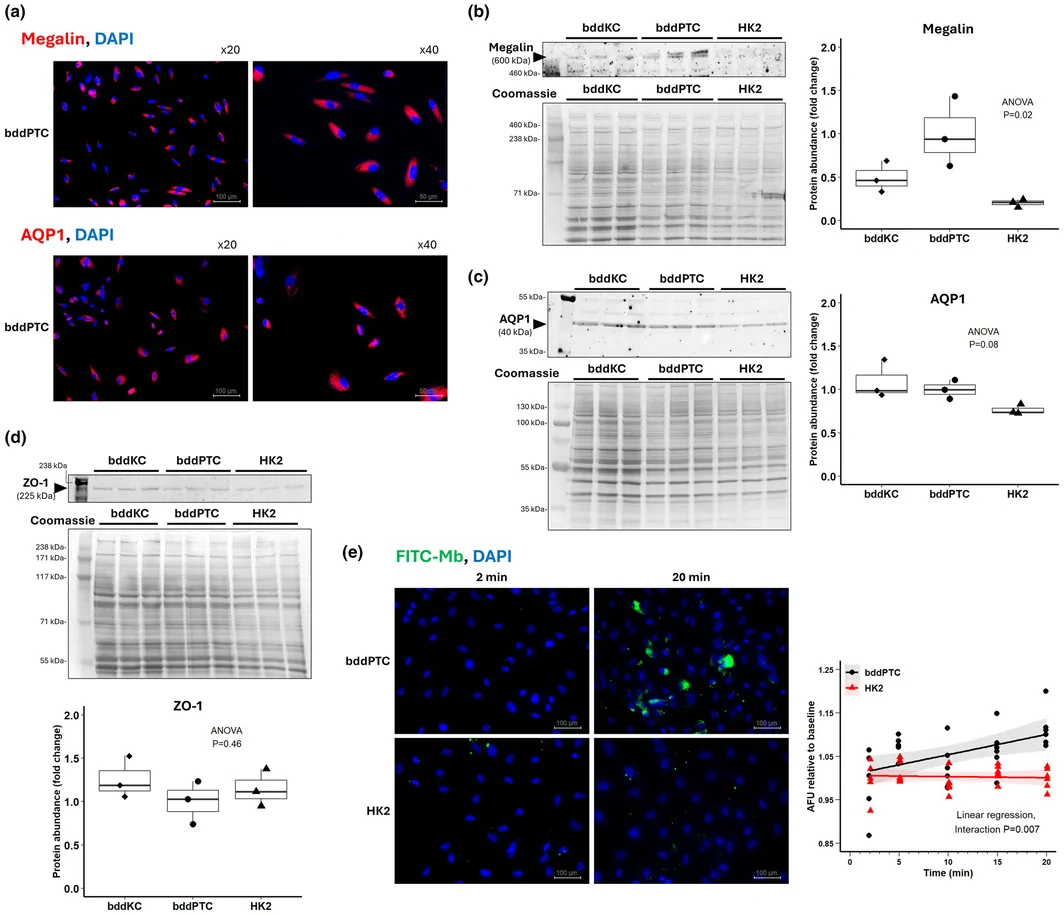
bddPTC express megalin and aquaporin‐1 and uptake myoglobin. (a) Immunofluorescence for megalin and aquaporin‐1 (AQP1) in bddPTC. Nuclei were stained with DAPI. Magnification: ×20 or ×40. Scale bar: 100 or 50 μm. (b–d) Immunoblotting for megalin, AQP1, and ZO‐1 in bddKC, bddPTC (at passage 2), and HK2 cells. The expressions were standardized to total protein amount with Coomassie staining. Expression value is shown as fold change to the bddPTC group average. Difference between the groups was assessed with one way‐analysis of variance (ANOVA) followed by Tukey's post hoc analysis. (e) bddPTC and HK2 cells were incubated with fluorescein isothiocyanate‐conjugated myoglobin (FITC‐Mb). Magnification: ×20. Scale bar: 100 μm. Fluorescent change was evaluated to assess cellular uptake of myoglobin. Differences in fluorescent slope between groups were assessed as interaction of groups with incubation time with linear regression model. AFU, arbitrary fluorescence unit.

To assess whether bddPTC sustain PT functional characteristics, expressions of megalin and AQP1, another PT marker (Venkatesan et al., 2026), and endocytic function were analyzed. Immunocytochemistry demonstrated that a significant portion of bddPTC (*n* = 4) retained protein expression of megalin (87.2% ± 7.7% at passage 2 and 88.4% ± 2.2% at passage 3) and AQP1 (74.2% ± 7.6% at passage 2 and 67.9% ± 3.8% at passage 3) (Figure 2a). Immunoblot analysis was performed on bddKC, bddPTC, and HK2 cells, a human cell line which has documented loss of megalin expression (Khundmiri et al., 2021) (*n* = 3/group). The analysis demonstrated that bddPTC megalin was nearly double that of bddKC (0.49 ± 0.18 compared to bddPTC, *p* = 0.11) and five times that of HK2 cells (0.20 ± 0.04, *p* = 0.02) (Figure 2b). Our reanalysis of publicly‐available data (Khundmiri et al., 2021) demonstrated that HK2 cells exhibited similar *LRP2* mRNA levels compared to other human PT cell lines (relative expression; HK2 cells 0.81 ± 1.15, ANOVA *p* = 0.87) (Figure S2). Megalin expression in bddPTC was sustained through until passage 4 (Figure S3). AQP1 expression was assessed with immunoblotting, and the expression level tended to be higher in bddPTC and bddKC (1.09 ± 0.22 compared to bddPTC) relative to HK2 cells (0.76 ± 0.06), though the difference among the groups was statistically insignificant (ANOVA *p* = 0.08) (Figure 2c). ZO‐1, tight junction protein and a marker of differentiated epithelia (Legouis et al., 2015), was detected in bddPTC by immunoblotting and the protein abundance of ZO‐1 in bddPTC was similar to bddKC and HK2 cells (relative expression; bddKC 1.26 ± 0.24, HK2 cells 1.15 ± 0.22, ANOVA *p* = 0.46) (Figure 2d). To test endocytic function, bddPTC and HK2 cells were incubated with FITC‐Mb for 20 min. bddPTC took up FITC‐Mb more than HK2 cells (Increasing rate of FITC‐fluorescence; 0.0047 ± 0.0016 Δarbitrary fluorescence units [ΔAFU]/min in bddPTC and − 0.0003 ± 0.0008 ΔAFU/min in HK2 cells, *p* = 0.007, Figure 2e).

### 3.3 Myoglobin uptake in bddPTC was decreased by inhibitors for megalin and endocytosis

**FIGURE 3:**
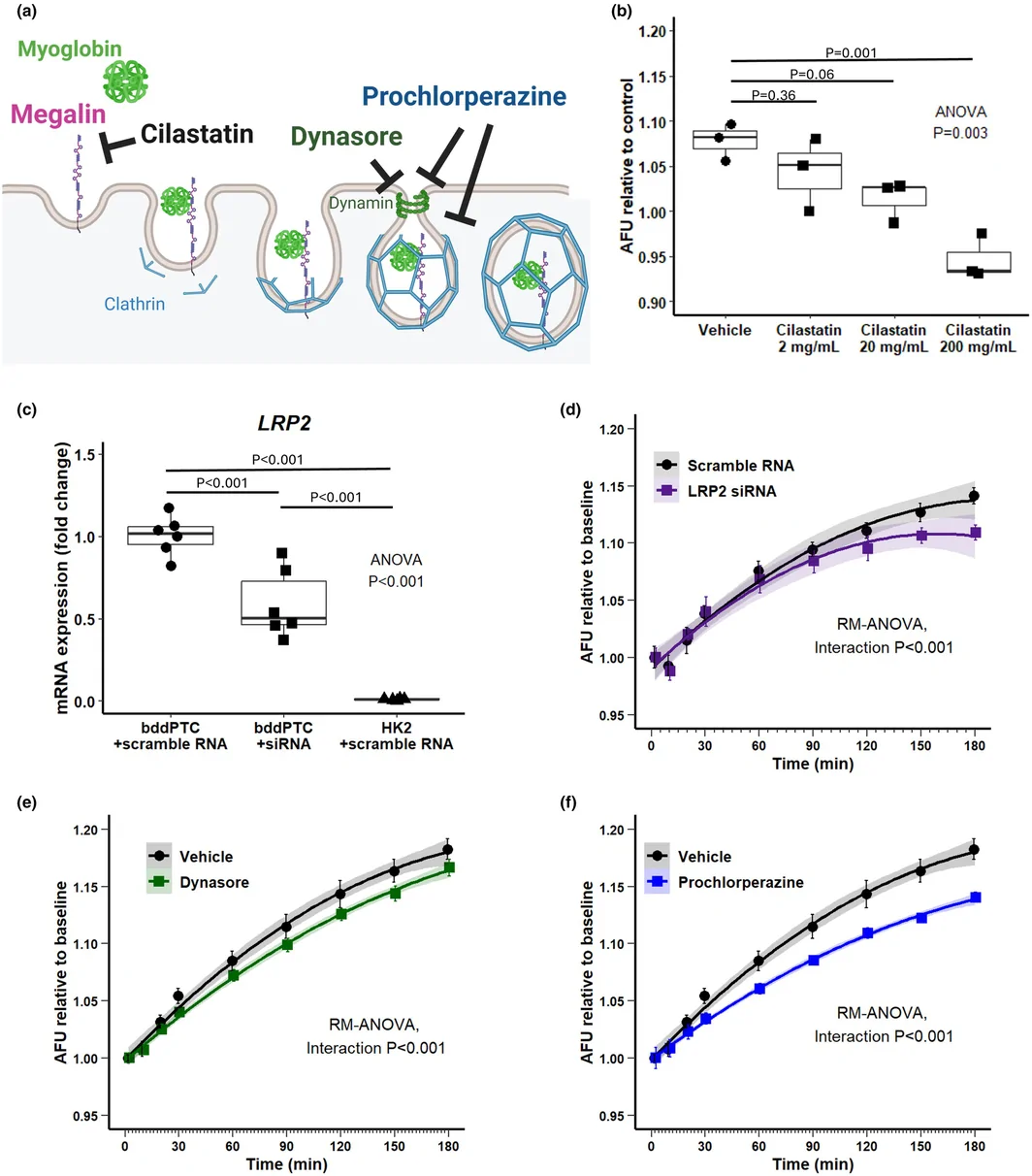
Endocytosis inhibitors decreased uptake of myoglobin in bddPTC. (a) Schematics of different components of endocytosis. Myoglobin binds to megalin and is transferred into the cell via endocytosis. Cilastatin blockades megalin. Dynasore inhibits dynamin, which facilitates cargo detachment from the plasma membrane. Prochlorperazine inhibits dynamin and clathrin. (b) bddPTC treated with cilastatin (2, 20, or 200 mg/mL) or vehicle were incubated with FITC‐Mb. Fluorescence change was assessed 15 min after incubation. Differences between groups were assessed with one‐way ANOVA followed by Dunnet's post hoc analysis. (c) bddPTC and HK2 were treated with small interfering RNA (siRNA) targeting *LRP2* or scramble RNA. *LRP2* mRNA expression is shown as fold change to the average expression in the scramble RNA‐treated bddPTC group. Differences between the groups were assessed with one‐way ANOVA followed by Tukey's post hoc analysis. (d–f) bddPTC treated with siRNA targeting *LRP2*, dynasore (10 μM) or prochlorperazine (100 μM) were incubated with FITC‐Mb. Differences in fluorescence change between the groups were assessed as the interaction of the groups with incubation time with repeated measurement ANOVA in (d–f).

To test the functional capacity of bddPTC endocytic machinery, myoglobin uptake was assessed in bddPTC pre‐treated with *LRP2*‐directed siRNA or endocytocis inhibitors. A schematic of the different components of endocytic mechanisms is shown in Figure 3a. Pre‐treatment with the megalin inhibitor cilastatin reduced myoglobin uptake at 20 min in a dose‐dependent fashion (Figure 3b). When bddPTC were treated with siRNA targeting *LRP2* (the gene encoding megalin), *LRP2* transcript was suppressed by 50% (Figure 3c). While *LRP2*‐directed siRNA did not decrease the uptake of myoglobin at 20 min relative to scramble siRNA, myoglobin uptake was decreased at 180 min in *LRP2*‐directed siRNA‐treated bddPTC cells relative to scramble siRNA‐treated cells (Figure 3d). Dynasore (10 μM) and prochlorperazine (100 μM) both caused reduction in uptake of myoglobin at 180 min (Figure 3e,f).

### 3.4 VCAM1 expression in bddPTC

**FIGURE 4:**
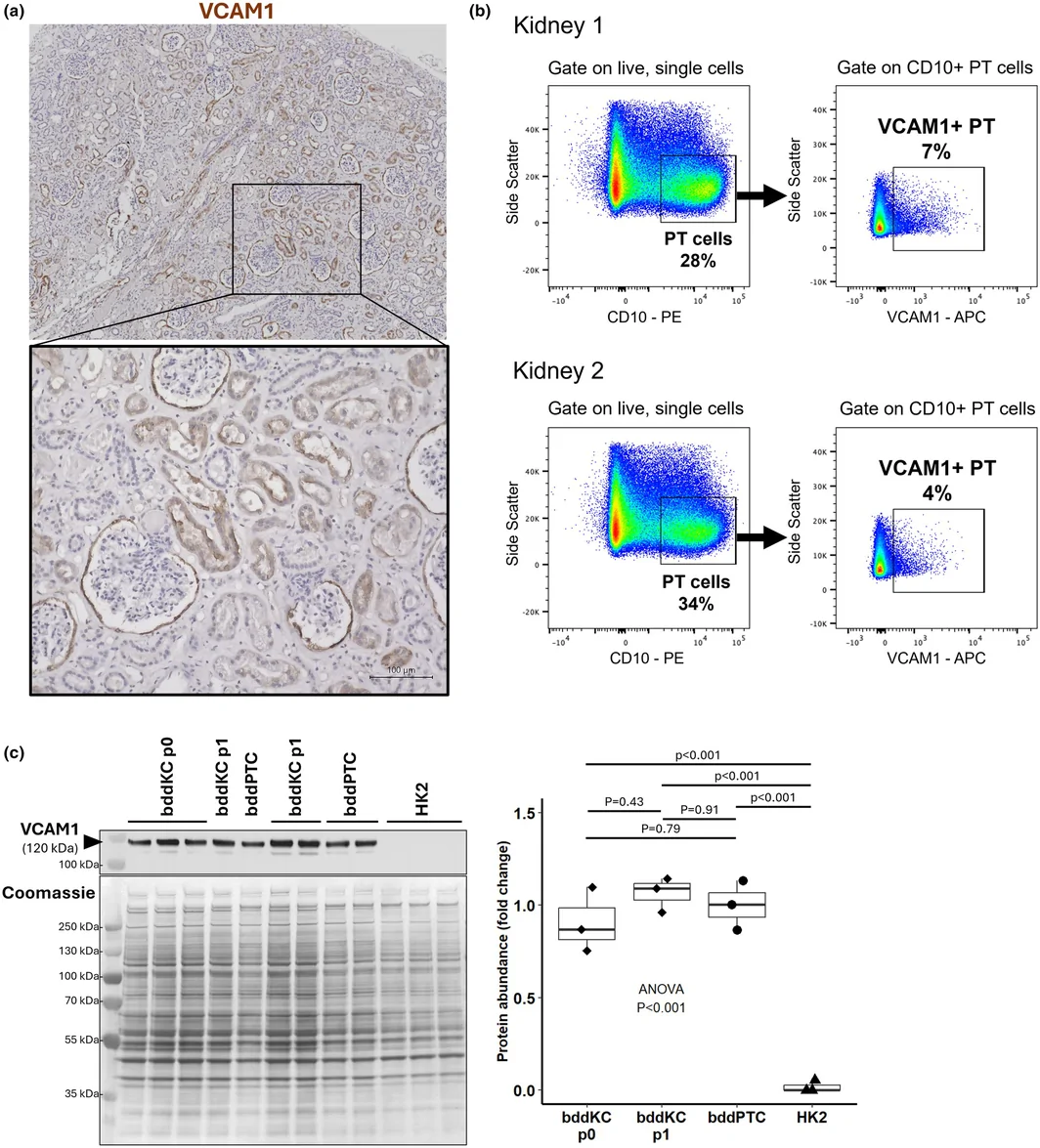
Sustained expression of VCAM1 in bddPTC. (a) Immunohistochemistry for VCAM1 in donor kidney, demonstrating many VCAM1+ proximal tubules (PT). Magnification: ×10 in the upper panel and ×20 in the lower panel. Scale bar: 100 μm. (b) bddKC were analyzed with flow cytometry. 30% of bddKC consist of PT cells and 5% of PT cells are positive for VCAM1. (c) Immunoblotting for VCAM1 in bddKC, bddPTC (passage 2), and HK2 cells. Expression level of VCAM1 was standardized with total protein amount quantified with Coomassie staining. Expression value is shown as fold change to the average in bddPTC group. Differences between the groups were assessed with one‐way ANOVA followed by Tukey's post hoc analysis. APC, allophycocyanin; PE, phycoerythrin; VCAM1, vascular cell adhesion molecule‐1.

Since we observed acute tubular necrosis in the donor kidney, we hypothesized that a portion of isolated bddPTC would resemble injured PT cells in vivo. We tested this hypothesis by assessing expression of VCAM1, a marker for “failed repair” PT cells that exhibit maladaptive functions such as pro‐fibrotic transcript expression and pro‐inflammatory cytokine production (Kirita et al., 2020; Muto et al., 2021), in donor kidney sections and bddPTC. Immunohistochemistry revealed that VCAM1 was abundantly expressed in the tubules of the donor kidney (Figure 4a). Flow cytometry analysis demonstrated that 4%–7% of CD10/CD13‐positive PT cells in the donor kidney were also positive for VCAM1 (Figure 4b and Figure S4). Immunoblot analysis demonstrated that VCAM1 expression was sustained in cultured bddKC and bddPTC, about 50 times higher compared to HK2 cells (relative expression; bddKC at passage 0 0.91 ± 0.17, bddKC at passage 1 1.06 ± 0.09, bddPTC 1.00 ± 0.02, HK2 cells 0.02 ± 0.01, ANOVA *p* < 0.001) (Figure 4c). Interestingly, analysis of publicly available data (Khundmiri et al., 2021) demonstrated *VCAM1* mRNA levels in HK2 cells were relatively higher than other human PT cell lines (relative expression; HK2 cells 2.18 ± 0.07, ANOVA *p* = 0.04) (Figure S5).

## 4 DISCUSSION

The most important contribution of this study is a robust and reproducible protocol for isolation and culture of human PT cells from brain‐dead donor kidneys. This technique generates a robust yield of human PT cells that retain physiologic functions, as evidenced by sustained megalin and aquaporin‐1 expression throughout passaging, and retained endocytosis capacity. Megalin is a multifunctional receptor expressed on the apical cell surface of PT. The extracellular motif of megalin binds to a wide range of substances in glomerular filtrate (Eshbach & Weisz, 2017; Goto et al., 2023; Mukherjee et al., 1997). Once megalin binds to targets, clathrin within the cell triggers a plasma membrane invagination which entrains cargo inside the cells, engulfing the substrate (Eshbach & Weisz, 2017). The cargo is ultimately detached from the plasma membrane by dynamin. Through these processes, PT cells recover many filtered substances, including myoglobin, albumin, hormones, and drugs (Eshbach & Weisz, 2017; Gburek et al., 2003; Hebert et al., 2023); this process is central to PT function and vital to organism homeostasis. Therefore, intact PT megalin‐mediated endocytosis is a critical benchmark for in vitro models of PT function. AQP1 is a major water transporter strongly expressed in PT, which plays a pivotal role in controlling water reabsorption and urine osmolality (Venkatesan et al., 2026). ZO‐1 is a scaffold protein of tight junction and a marker of polarized and differentiated epithelial cells (Legouis et al., 2015). The sustained expression of megalin, AQP1, and ZO‐1 and megalin‐mediated endocytosis of bddPTC together support the feasibility of this in vitro model for PT functional studies.

Although several human PT cell lines are commercially available, studies report these cell lines lose many physiologic functions (An et al., 2023; Khundmiri et al., 2021). Khundmiri, et al. analyzed transcriptomes of major human PT cell lines, including HK2 cells, and reported that none of those cell lines expressed *LRP2* relative to murine kidney tissue or primary cultured cell. It was also reported that HK2 cells and other (non‐human) PT cell lines weakly express proteins associated with endosomal and lysosomal functions (crucial processes following endocytosis) relative to isolated murine PT cells (Berquez et al., 2021). Additionally, while human PT cells isolated from living patients expressed PT‐defining genes, RPTEC‐TERT1, a human PT cell line, lacked or had substantially reduced expression of many these genes (Zhao & Imperiale Michael, 2019). Consistent with these previous reports, we demonstrate that HK2 cells lacked megalin expression and had decreased endocytosis capacity compared to bddPTC.

Human PT cells can be isolated from living patients, and several studies present protocols to isolate the cells from the normal‐appearing part of surgically‐resected malignant kidneys or from kidney biopsy specimens (Aceves et al., 2022; Presnell et al., 2011; Vesey et al., 2009). Human PT cells isolated with these protocols reportedly sustain PT physiological functions (Presnell et al., 2011; Vesey et al., 2009; Zhao & Imperiale Michael, 2019). However, yield may be limited, and additional limitations are potential contamination from malignant cells or phenotypic change across all cells induced by the pro‐oncogenic environment (McEvoy et al., 2022; Yaigoub et al., 2022). Ethical compliance and acquisition may be challenging when working with samples from living patients, and because nephrectomy samples are not perfused or cooled for transplantation, rapid cell isolation and deidentification may be difficult to achieve (Danačíková et al., 2024). Variations in hospital/research center requirements and organization may present challenges when using living‐donor tissue sources. Conversely, organ procurement for transplant is governed by national and international regulations, with broadly consistent processes and organizations for authorization, organ procurement, and research designation (Silva et al., 2024). An ethical imperative of organ transplant is to minimize waste of the donor's gift of organs; therefore, an additional benefit of research use of research‐designated donor organs is benefit to human knowledge from kidneys which would otherwise be discarded. Primary culture of murine kidney cells is also often used in physiologic investigation (Ding et al., 2018; Kamiyama et al., 2012; Terryn et al., 2007). However, metabolic profiles, gene expressions, and physiological functions of PT differ between mice and humans (Rydell‐Törmänen & Johnson, 2019). Therefore, we anticipate this work will expand research opportunities using human PT cells in vitro and contribute to progress in understanding of human PT function.

We demonstrated VCAM1 expression on donor kidney PT cells, which was sustained in bddPTC after culturing. During AKI recovery, a subset of injured PT cells assumes a unique persistent proinflammatory and profibrotic phenotype (Kirita et al., 2020; Muto et al., 2024; Xu et al., 2025). These cells are called “maladaptive” or “failed repair” PT cells, and are believed to play a crucial role in the AKI to CKD transition. VCAM1 is not constitutively expressed in healthy PT and has been identified as a marker of those “failed repaired” PT cells (Kirita et al., 2020). Brain‐dead donors are in a distinct pathophysiological condition, which can include hypotension, intravascular coagulation, cardiac arrhythmia, and metabolic acidosis (Au & Bell, 2014). Damman, et al. reported that the gene expression associated with hypoxia, complement, and coagulation pathways is upregulated in the kidney of brain‐dead donors compared to healthy living donors (Damman et al., 2015). Additionally, Sally, et al. demonstrated a >3‐fold increase in kidney VCAM1 mRNA 6 h after induction of brain death in a porcine model (Sally et al., 2018). Therefore, it seems likely that brain death may cause kidney injury with failed repair of donor kidney and sustained expression of VCAM1 in bddPTC. An alternative, though less likely explanation is that high VCAM1 expression and acute tubular necrosis in a donor kidney may be due to prolonged ischemia time after recovery. While the present study demonstrated that bddPTC strongly express VCAM1 protein, about 50 times higher compared to HK2 cells, mRNA expression of VCAM1 in HK2 cells is relatively higher than that in other human cell lines, according to our reconstructive analysis of the ESBL database. Therefore, bddPTC provide a distinct culture model of human PT cells enriched for expression of VCAM1, possibly a useful in vitro model for investigations into PT failed repair. However, the significance of VCAM1 expression in cultured kidney cells is still unknown. Telang, et al. isolated PT cells from mouse kidneys and reported that the expression of failed repair‐associated genes continuously increased with culturing to day 14 along with the increase in *Vcam1* expression until day 7 (Telang et al., 2024). Muto, et al. reported sustained expression of VCAM1 in RPTEC, a commercially available primary human PT cell product (Muto et al., 2021). Additionally, they reported scattered expression of VCAM1 in the human kidney without kidney injury, suggesting that some failed repair occurs constitutively in healthy kidneys. Taken together, these data do not determine the source of strong VCAM1 expression in bddPTC; it may be due to primary culture itself, or to failed repair of the donor kidney, or to some other factor. Further studies are desired to clarify this issue.

Our study has limitations. First, donor demographics are unknown. Demographic factors including age and sex, underlying diseases, and cause of death may influence the function and phenotypes of bddPTC. All brain‐dead donors have suffered critical illness, and some component of acute kidney injury is likely present (Kotloff et al., 2015). Management of brain‐dead donors is complex and includes administration of intravenous fluids, vasoconstrictors, and endocrine mediators such as thyroid hormone to mitigate organ injury (Swanson, Adams, et al., 2020; Swanson, Patel, et al., 2020). Additionally, human subjects protections in our protocol required blinding to warm ischemic time (the time between renal artery clamping and kidney cooling after removal from the donor). This important variable may likely modulate the phenotypic features of bddPTC. A comparison of bddPTC to primary‐cultured PT cells from non‐brain‐dead donors might help understand this issue, although controlling warm ischemia time in this setting is complex. Second, physiologic functions of bddPTC other than endocytosis of myoglobin were not assessed. It is unknown whether other PT functions, such as water and sodium transport, are sustained in bddPTC. Lastly, we cultured bddPTC in two dimensional (2D) conditions and have not assessed three dimensional (3D) culture or shear‐stress inducing conditions. Although 2D culture is the most common and established method, recent studies suggest that 3D culturing on a specific scaffold architecture preserves phenotypes of culture PT in vivo (Aceves et al., 2022; Hoppensack et al., 2013) and shear‐stress induction augments endocytic function (Lackner et al., 2024). Further research with 3D culture and shear‐stress induced bddPTC is warranted.

In conclusion, brain‐dead donor‐derived PT cells are a feasible platform for studies of PT function which retain endocytic function.

## AUTHOR CONTRIBUTIONS

**Daiki Aomura:** Data curation; formal analysis; investigation; methodology; project administration. **Kevin G. Burfeind:** Data curation; formal analysis; methodology; project administration. **Yoshio Funahashi:** Conceptualization; formal analysis; investigation; methodology; project administration. **Tahnee Groat:** Funding acquisition; project administration; resources. **Adam C. Munhall:** Methodology; project administration. **Darren J. Malinoski:** Supervision. **Michael P. Hutchens:** Conceptualization; funding acquisition; investigation; methodology; project administration; resources; supervision.

## FUNDING INFORMATION

This work was supported by the US Department of Veterans Affairs Merit Review Award# I01BX04288 (to M.P.H.) This material is the result of work which was supported with resources and the use of facilities at the Portland Veterans Affairs Medical Center. The contents do not represent the views of the U.S. Department of Veterans Affairs or the United States Government.

## CONFLICT OF INTEREST STATEMENT

The authors have no conflicts to disclose.

## Supporting information


**Table S1:** The list of instruments and reagents.
**Figure S1:** Immunohistochemistry for LRP2 in brain‐dead donor kidney.
**Figure S2:** mRNA expression of *LRP2* in human cell lines.
**Figure S3:** Immunoblotting for LRP2 in bddPTC at through passage 4.
**Figure S4:** Flow cytometry gating strategy and controls.
**Figure S5:** mRNA expression of *VCAM1* in human cell lines.

## Data Availability

All code, raw data, and original full length blotting images used to perform analyses in this manuscript are available on the Hutchens lab GitHub (https://github.com/HutchensLab/Aomura_Burfeind_isolation_characterization).
