# Supplementary material for: Isolation and functional characterization of primary human proximal tubular epithelial cells from brain‐dead organ donors
Source: Physiol Rep. 2026 Jun 4;14(11):e70963. doi: 10.14814/phy2.70963 (PMC13581086; doi:10.14814/phy2.70963)
Supplement: Supplementary file 1 [file PHY2-14-e70963-s001.docx]

**Supplementary table S1:** The list of instruments and reagents

| Instruments | |
| --- | --- |
| - Scalpel | |
| - Scissors | |
| - Forceps | |
| - 100 mL beaker | |
| - Small or middle size petri dish (for mincing the kidney tissue at c-7) | |
| - Large size petri dish (for cell solution filtered through 250 μm strainer at c-13) | |
| - 70 μm cell strainer (Cat. No. 352350, Corning) - 100 μm cell strainer (Cat. No. 352660, Corning) - 250 μm stainless steel sieve (Cat. No. sz0000169, QuaFooLAB) - Syringe plunger - 50 mL tubes | |
| Reagents | |
| - Rat Collagen Type 1 (Cat. No. C3867, Sigma) | |
| - ITS Premix Universal Culture Supplement (Cat. No. 354351, Corning) | |
| - Fetal Bovine Serum (FBS) (Cat. No. F-0500-A, Atlas Biologicals) | |
| - Liberase^TM^ (Cat. No. 5401119001, Sigma) | |
| - Dulbecco's Modified Eagle Medium/Nutrient Mixture F-12 (DMEM/F12) (Cat. No. D8437, Sigma) | |
| - Penicillin-Streptomycin 10,000 U/mL (P/S) (Cat. No. 15140-122, Thermo Fisher Scientific) | |
| Mixture of reagents | Amount |
| Rat collagen type 1 solution |  |
| - Rat Collagen Type 1 - ddH2O | 500 μL  50 mL |
| DMEM/F12+1%P/S |  |
| - P/S - DMEM/F12 | 5 mL  500 mL |
| Digestion medium |  |
| - Liberase (26U/mL, dissolved in ddH2O) - DMEM/F12+1%P/S | 2 mL  200 mL |
| Culture medium-10% FBS |  |
| - FBS - DMEM/F12+1%P/S | 70 mL  630 mL |
| Culture medium-1%FBS |  |
| - FBS | 3 mL |
| - ITS Premix Universal Culture Supplement - DMEM/F12+1%P/S | 300 μL  300 mL |

**Supplementary figure S1. Immunohistochemistry for LRP2 in brain-dead donor kidney.**

Immunohistochemistry for LRP2 in donor kidney, demonstrating sustained LRP2 expression in proximal tubules. Magnification: x 10 in the left panel and x 20 in the right panel. Scale bar: 100 μm.

**Supplementary figure S2. mRNA expression of *LRP2* in human cell lines.**

*LRP2* mRNA in human PT cell in the Epithelial Systems Biology Laboratory database (<https://esbl.nhlbi.nih.gov/JBrowse/KCT/>). Expression levels were standardized by average expression levels of all 5 cell lines. Differences between groups were assessed with one way-ANOVA.

**Supplementary figure S3. Immunoblotting for LRP2 in bddPTC at through passage 4.**

Immunoblotting for megalin in bddPTC at passage 2, 3, and 4. The expressions were standardized to total protein amount with Coomassie staining. Expression value is shown as fold change to the average of passage 2 group. Difference between the groups were assessed with one way-ANOVA.

.

**Supplementary figure S4. Flow cytometry gating strategy and controls.**

A) Flow cytometry plots showing gating strategy used for Figure 4. B) Flow cytometry plots showing fluorescence minus one (all antibodies but VCAM1 antibody) controls used for gating strategy to identify VCAM1+ PT. All plots gated on Live, single cell, CD10+ PT. PE = phycoerythrin. PT = proximal tubule cell. VCAM1 = vascular cell adhesion molecule-1.

**Supplementary figure S5. mRNA expression of *VCAM1* in human cell lines.**

*VCAM1* mRNA in human PT cell in the Epithelial Systems Biology Laboratory database. Expression levels were standardized by average expression levels of all 5 cell lines. Differences between groups were assessed with one way-ANOVA followed by Dunnet’s post hoc analysis.
